# The Mouse MC13 Mutant Is a Novel ENU Mutation in Collagen Type II, Alpha 1

**DOI:** 10.1371/journal.pone.0116104

**Published:** 2014-12-26

**Authors:** Megan Cionni, Chelsea Menke, Rolf W. Stottmann

**Affiliations:** 1 Division of Human Genetics, Department of Pediatrics, Cincinnati Children's Hospital Medical Center, Cincinnati, Ohio, 45229, United States of America; 2 Division of Developmental Biology, Department of Pediatrics, Cincinnati Children's Hospital Medical Center, Cincinnati, Ohio, 45229, United States of America; Duke University Medical Center, United States of America

## Abstract

Phenotype-driven mutagenesis experiments are a powerful approach to identifying novel alleles in a variety of contexts. The traditional disadvantage of this approach has been the subsequent task of identifying the affected locus in the mutants of interest. Recent advances in bioinformatics and sequencing have reduced the burden of cloning these ENU mutants. Here we report our experience with an ENU mutagenesis experiment and the rapid identification of a mutation in a previously known gene. A combination of mapping the mutation with a high-density SNP panel and a candidate gene approach has identified a mutation in *collagen type II, alpha I (Col2a1)*. *Col2a1* has previously been studied in the mouse and our mutant phenotype closely resembles mutations made in the *Col2a1* locus.

## Introduction

Forward genetics in the mouse has been demonstrated to be an efficient tool for taking an unbiased approach to generate novel alleles in genes important for a wide range of functions, including organogenesis. These alleles represent powerful models of human development and disease. Multiple laboratories have shown the power of ENU mutagenesis over the past several years. These screens were initially aimed at general morphological and organogenesis defects [1], [2]. More recent experiments have been more focused on specific aspects of mouse development [3]–[5] or physiology [6]. A significant hurdle traditionally associated with forward genetics has been the attendant task of identifying the causal mutation leading to the phenotype of interest. Positional cloning with a combination of meiotic recombination mapping and a candidate gene sequencing approach has often been successful [3], [7]. In this report, we describe our cloning of a novel mutant allele of *collagen type II, alpha I (Col2a1)*.

## Results and Discussion

### ENU mutagenesis screen for congenital organogenesis phenotypes

**Figure 1:**
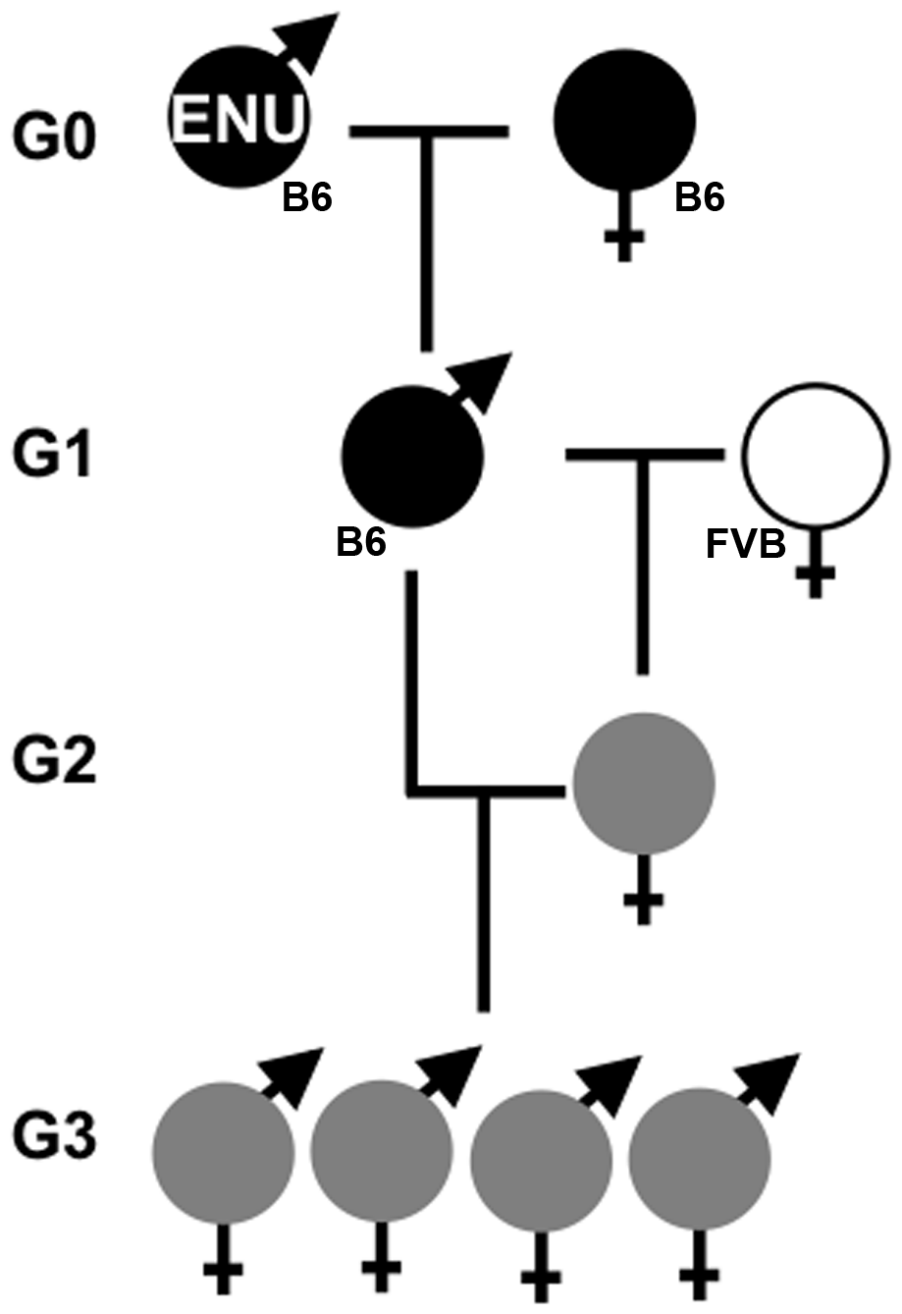
A three-generation breeding scheme to identify recessive ENU mutations affecting organogenesis. Generation 0 (G0) B6 males are treated with ENU and mated to untreated B6 females to create G1 males. Each G1 establishes an independent pedigree and is mated with FVB females (white) to create G2 females. The resulting G2 females are potential heterozygous carriers of an ENU mutation and obligate heterozygotes at all FVB and B6 SNPs (grey). These G2 females are backcrossed to the G1 and sacrificed to analyze the G3 generation at E18.5. B6 = C57BL/6J; FVB = FVB/NJ.

We recently performed an ENU mutagenesis experiment in the mouse as part of our continuing efforts to understand the genetic basis of congenital malformations affecting the forebrain and craniofacial tissues. Similar to our previous efforts, this breeding strategy included a genetic outcross to combine a genetic mapping component with our exome analysis [7], [8]. We began with mutagenized C57BL/6J (B6) G1 males donated from an ENU mutagenesis project at the Jackson Laboratories. These males are the progeny of B6 males which were subjected to a standard ENU mutagenesis protocol and then mated to untreated B6 mice to generate the G1 males. We then mated these G1 males to wild-type FVB/NJ (FVB) mice to generate the G2 offspring. The G2 females were mated back to the G1 males to create the G3 generation, which may be potentially homozygous for any ENU alleles present (Fig. 1). As in previous experiments, we harvested the G3 embryos at embryonic day (E) 18.5 with the goal of recovering mutations that might result in severe organogenesis phenotypes which would otherwise be lethal after birth.

### Line MC13 mutants have multiple skeletal phenotypes

**Figure 2:**
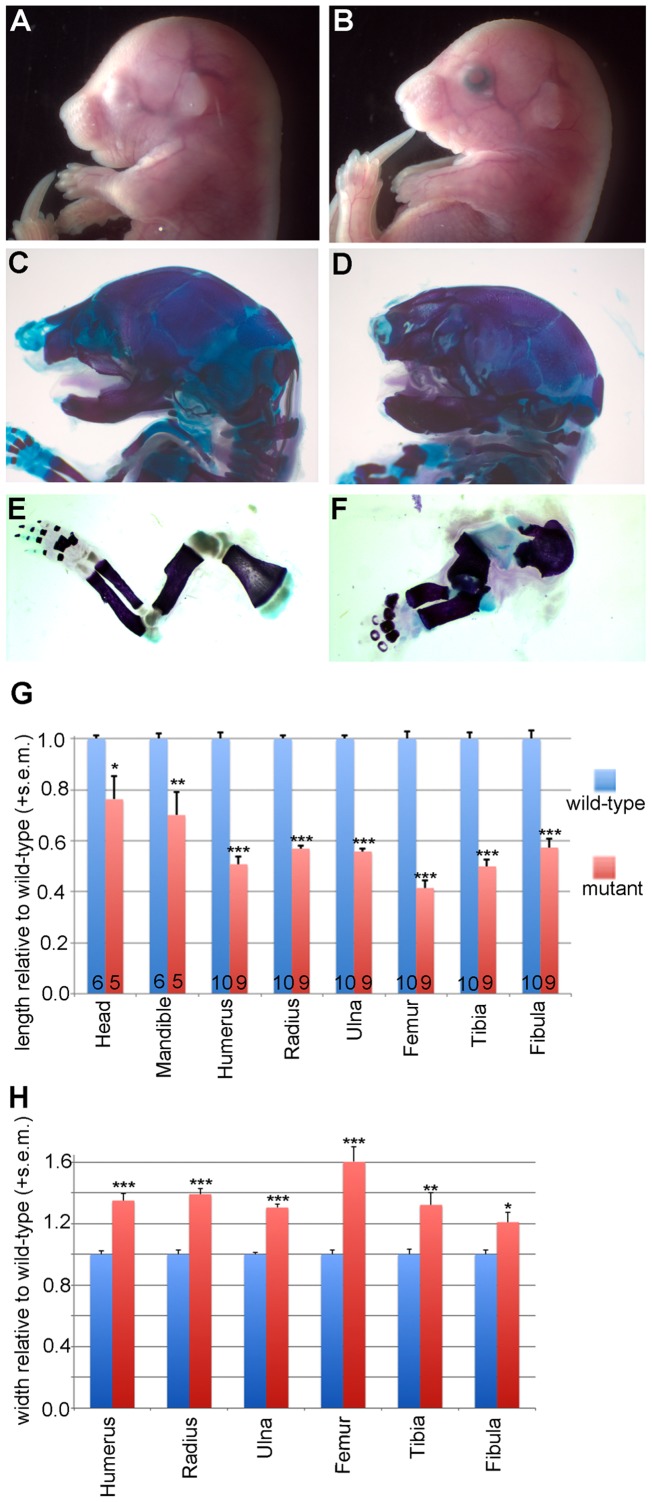
MC13 phenotypes. Mutant embryos (B) are grossly distinguishable from wild-type littermates (A) by the shape of their heads and shortened, thickened limbs. (C–F) Skeletal preparations to highlight bones (red) and cartilage (blue) show the dramatic skeletal phenotypes in MC13 mutants (D,F). (G,H) Measurements of heads and limbs show significant shortening (G) and widening (H) of these elements in MC13 mutants (sample size indicated for each bone. *p<0.02, **p<0.006, ***p<0.0001).

We recovered one particularly striking mouse mutant phenotype which we report here. *MC13* mutants were readily identifiable from control littermates by their abnormally shaped heads and limbs at E18.5 (Fig. 2B). We dissected out brain tissue from mutants and noted a mild microcephaly phenotype in some mutants but a normally patterned brain. In order to highlight the skeletal elements, we performed bone and cartilage stains. These skeletal preparations revealed dramatic phenotypes in multiple skeletal elements of mutant embryos as compared to wild-type littermates (Fig. 2C–F). The heads of mutant embryos were smaller and the mandibles were shorter as compared to control littermates. We also noted all long bones examined were significantly shorter and wider than controls. At E18.5 measurements were taken from multiple skeletal elements and all of these differences were statistically significant (Fig. 2G,H).

### Cloning of the MC13 mutants

**Figure 3:**
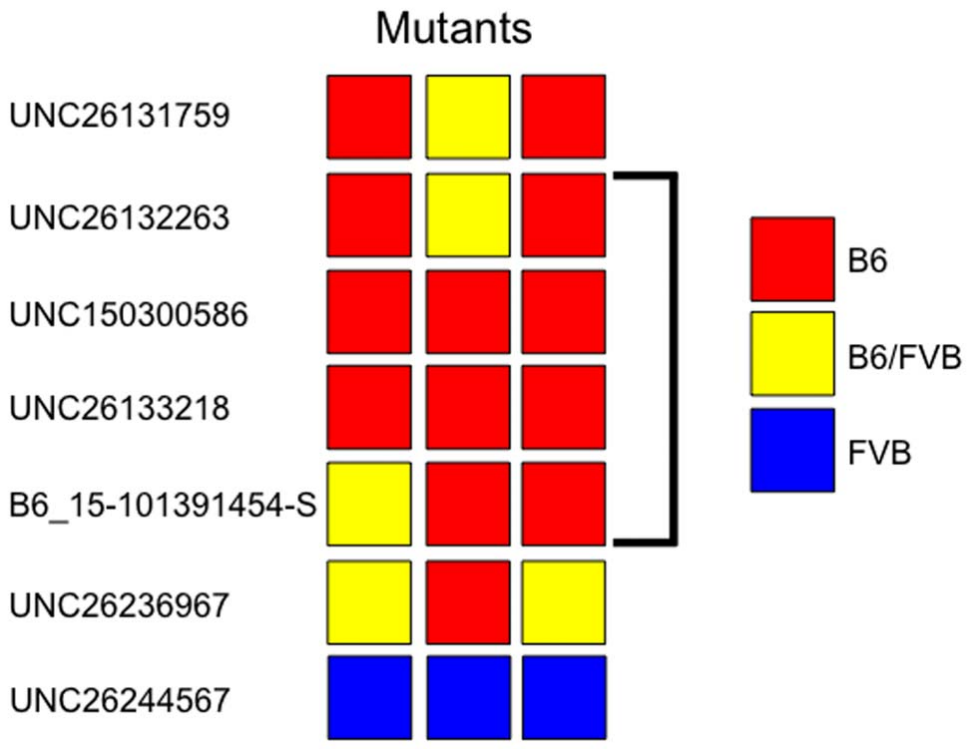
SNP mapping of the MC13 mutants. A whole-genome SNP panel indicates a region of homozygosity among three MC13 mutant samples. SNPs matching the B6 genome are indicated in red, FVB in blue and heterozygous in yellow. The three mutants show a B6 (ENU-treated) haplotype between SNPs UNC26132263 and B6_15-101391454-S.

In order to identify the causal mutation in the MC13 mutants, we performed single nucleotide polymorphism (SNP) mapping across the mouse genome. We submitted three mutant DNA samples to the MEGAMUGA mouse SNP panel. This is a commercial panel of 77,800 SNPs spaced throughout the genome at an average distance of 33 Kb. Given the ENU treatments were administered to the B6 mice (Fig. 1), we followed the standard approach of looking for regions of the genomes in which all three mutants were homozygous for the B6 marker: a B6 haplotype. Note however, the cross to create the G1 males was between B6 mice. This did introduce some B6 genomic material not strictly associated with the ENU mutagenesis. With the three mice selected for mapping, we identified a single 8.2 Mb region of B6 homozygosity (Fig. 3). This interval was defined at either end by heterozygosity for the SNPs UNC26132263 (chr15: 93,158,312, Bld37) and B6_15-101391454-S (chr 15: 101,391,454). Upon mapping to the mouse genome, this region was found to contain 122 protein-coding genes in the MGI database.

**Figure 4:**
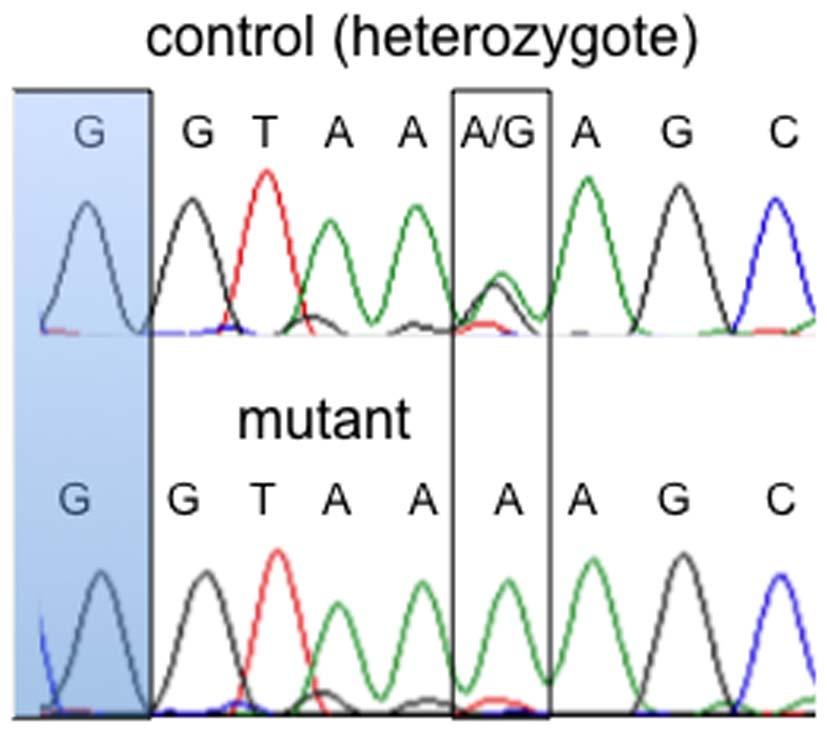
Sanger sequencing of an ENU mutation at the *Col2a1* locus. A heterozygous sample shows the wild type (G) and ENU mutant allele (A). Mutant DNA is homozygous for the ENU variant (A). The ENU mutant locus is boxed and the exon 21 sequence is indicated in blue.

A survey of known phenotypes in the region showed a high similarity between our MC13 mutant and previous ablations of *Col2a1*. Sequencing of the *Col2a1* locus (ENSMUSG00000022483) identified a mutation (G>A) in the intron between exons 21 and 22, five nucleotides from the end of exon 21 (Fig. 4). We identified this mutation to be present in multiple mutants with Sanger sequencing and absent from phenotypically normal embryos (a heterozygote is shown in Fig. 4). We next developed a custom Taqman SNP assay for this mutation. A survey of 104 embryos from 10 litters aged E16.5 to E18.5 indicated that all phenotypic mutants were homozygous for the G>A change. Furthermore, no adult animals in more than 75 genotyped were homozygous for the variant, and all phenotypic mutants came from animals heterozygous for the variant. We therefore conclude that the MC13 phenotype is most likely due to this mutation in *Col2a1*.

### The MC13 mutation in Col2a1 causes an incompletely penetrant splicing defect

**Figure 5:**
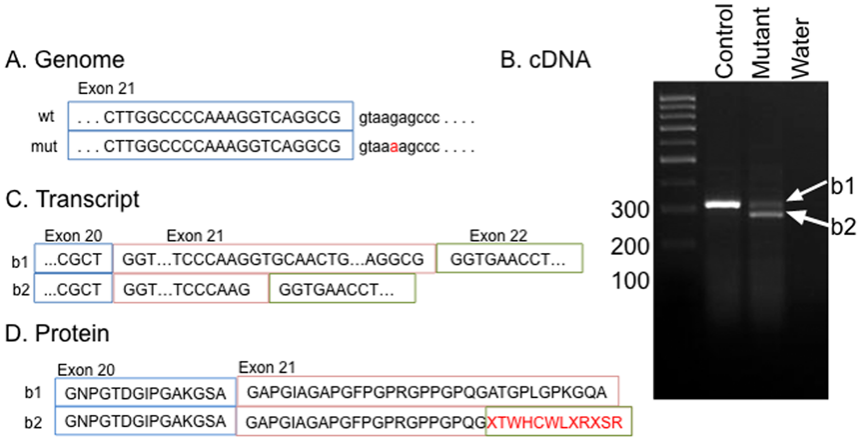
The MC13 mutations alters splicing of *Col2a1*. (A) The ENU mutation is in the intron after exon 21 (red). (B) Analysis of the *Col2a1* cDNA transcript in this area of the gene indicates an abnormal splice variant in the mutant sample (b2) in addition to the expected PCR product (b1). (C) The b2 PCR product represents an mRNA that does not include all of exon 21, but prematurely transitions to exon 22. (D) The abnormal transcript codes for a premature stop codon in exon 21 which is predicted to translate a protein approximately 30% the length of wild-type.

The location of the identified variant immediately downstream of an exon/intron boundary led us to examine the *Col2a1* transcript (ENSMUST00000023123) in MC13 mutants (Fig. 5A). RNA was purified and *Col2a1* cDNA was synthesized *in vitro* with a gene specific primer. PCR reactions for the coding region surrounding the mutation and possibly affected by a splicing defect were performed. Consistent with a splicing defect, the mutant cDNA shows two discrete PCR amplification products (Fig. 5B). We sequenced all PCR products and the wild-type band corresponds to the expected transcript sequence (Fig. 5C). The mutant cDNA shows a preferential amplification for a smaller PCR species (Fig. 5B). This represents a truncation of exon 21(Fig. 5C) and splicing into exon 22. This alternate transcript codes for a protein with a premature stop codon just after exon 21 (p. A445X, Fig. 5D), thus generating a predicted protein approximately 30% of the length of wild-type protein (normally 1,487 amino acids). We were not able to identify any antibodies raised against an epitope specific to *Col2a1* and appropriate to test for production of this truncation *in vivo*. We performed semi-quantitative analyses of the transcript levels between wild-type and mutant. Multiple experiments showed the total amount of *Col2a1* transcript in mutants was 65–80% of wild-type controls. The mutant form comprised 60% of the total mutant cDNA. We were unable to generate the predicted mutant protein *in vitro* to assess its translation and/or stability, likely because of the high GC-content surrounding the mutant splice site between exon 21 and 22.

### MC13 mutants phenocopy Col2a1 mutants

We originally identified *Col2a1* as a candidate gene for the MC13 mutation based on multiple similarities between the ENU mutant and the published null phenotypes. The most obvious of these phenotypes is the skeletal defects. Transgenic mice expressing an intragenic deletion of *Col2a1* as well *Col2a1* null mutants have been generated [9], [10]. In both cases, perturbation of the *Col2a1* locus leads to frequent perinatal lethality and skeletal phenotypes (e.g., short and thick limbs) similar to those we describe here in our MC13 mutants. The random nature of ENU mutagenesis does raise the possibility that another, closely linked, gene may theoretically be mutated to cause this phenotype. However, the phenotypic resemblance of our mutants to known *Col2a1* alleles, the 100% concordance between the *Col2a1* mutant genotype and phenotype to date, and the predicted effects of this mutation lead us to conclude this is indeed the most likely causal mutation. The definitive proof of this would be a complementation test between our MC13 allele and known allele of *Col2a1*. We, however, believe the evidence for this being the causal mutation is strong enough that we have not conducted this experiment for formal proof of our model.

### Human diseases caused by mutations in COL2A1

*COL2A1* mutations in humans have been identified in a number of skeletal dysplasias (for review, see [11]). A more comprehensive study of *COL2A1* mutations in human genetics shows mutations can lead to a wider range of disabilities than previously appreciated and can include joint disease that is not diagnosed until adulthood. Skeletal phenotypes may range from severe achondrogenesis and perinatal lethality to Stickler Syndrome [11], [12]. Most mutations leading to the chondrodysplasias are substitutions at a glycine residue within the triple helical domain which then interfere with multimer assembly [13], [14]. Multiple mutations coding for premature stop codons, similar to our mouse model, have been identified in Stickler Syndrome (e.g., [15]–[19]). We have not examined the heterozygous mice in our colony to see if they have any of the ocular phenotypes of Stickler patients as has been done for other models [20]. The phenotype in our homozygous mouse mutants is more similar to the null mouse phenotype and the severe human skeletal disorders than the features of Stickler Syndrome. A review of the literature suggests that premature truncations in collagen genes as seen in our ENU mutant may trigger the nonsense mediated RNA decay mechanism to remove the aberrant transcript from the cell [21]. However, homozygous expression of a truncated form of *Col2a1* in the mouse has also led to phenotypes similar to those seen in our mutants [9]. This truncated form of collagen is thought to incorporate into a pro-collagen molecule but then prevent the proper folding into a triple helical multimer, thus potentially disrupting the function of wild-type collagen molecules (*Col2a1* and others). Given that our analysis of the mutant transcript does indicate the normal variant is produced at some level, we suggest that some aberrant RNA is likely degraded but truncated peptides are produced. These then ultimately prevent the normal production and function of collagen fibrils and recapitulate aspects of both the null phenotype [10] and the overexpression of a minigene construct [9]. In conclusion, we show here that our ENU mutagenesis experiments continue to generate novel alleles modeling human diseases. The MC13 mutation is most likely an intronic mutation coding for a truncated protein product from the *Col2a1* locus. This represents a new mouse model of human disorders associated with collagen mutations.

## Methods

### Animal Husbandry

This study was carried out in strict accordance with the recommendations in the Guide for the Care and Use of Laboratory Animals of the National Institutes of Health. The protocol was approved by the Institutional Animal Care and Use Committee of the Cincinnati Children's Hospital Medical Center (protocol numbers 1D05052 and IACUC2013-0068). All euthanasia and embryo harvests were performed after isoflurane sedation to minimize animal suffering and discomfort. Animal euthanasia was via cervical dislocation and thoracotomy.

### SNP Mapping and Next Generation Sequencing

SNP mapping on the MEGAMUGA panel was performed by the Neogen Corporation (Lincoln, NE).

### Embryo Analysis

Embryos were dissected, examined and photographed with a Zeiss Discovery.V8, Axiocam MRc5and AxioVision software. Skeletal preparations to highlight bone and cartilage were done according to standard, published methods [22]. Measurements of skeletal elements were done within the Axiovision software suite and tabulated with Excel. Student t-tests were performed to measure significance of skeletal measurements.

### Transcript Analysis

RNA was prepared from whole heads of E16.5 MC13 mutants and wild-type control littermates using the TRIZOL reagent and manufacturer's protocols (Life Technologies, Grand Island, NY). cDNA was prepared using the SuperScript III First Strand cDNA Synthesis kit (Life Technologies) and gene specific primer (TCGGCCCTCATCTCTACATC). Transcripts were PCR amplified with primers for exons 19 (F; GAAGGTGCTCAAGGTTCTCG) and exon 23 (R; ACCTCGTTTGCCTTCTTCAC) and Sanger sequencing was performed at the CCHMC DNA Sequencing and Genotyping Core Facility. Quantification of the cDNA was done with the GelAnalyzer 2010a software.
